# *Clock* Gene Variation Is Associated with Breeding Phenology and Maybe under Directional Selection in the Migratory Barn Swallow

**DOI:** 10.1371/journal.pone.0035140

**Published:** 2012-04-10

**Authors:** Manuela Caprioli, Roberto Ambrosini, Giuseppe Boncoraglio, Emanuele Gatti, Andrea Romano, Maria Romano, Diego Rubolini, Luca Gianfranceschi, Nicola Saino

**Affiliations:** 1 Dipartimento di Biologia, Università degli Studi di Milano, Milano, Italy; 2 Dipartimento di Biotecnologie e Bioscienze, Università di Milano-Bicocca, Milano, Italy; 3 Dipartimento di Scienze Biomolecolari e Biotecnologie, Università degli Studi di Milano, Milano, Italy; Pennsylvania State University, United States of America

## Abstract

**Background:**

In diverse taxa, photoperiodic responses that cause seasonal physiological and behavioural shifts are controlled by genes, including the vertebrate *Clock* orthologues, that encode for circadian oscillator mechanisms. While the genetic network behind circadian rhythms is well described, relatively few reports exist of the phenological consequences of and selection on *Clock* genes in the wild. Here, we investigated variation in breeding phenology in relation to *Clock* genetic diversity in a long-distance migratory bird, the barn swallow (*Hirundo rustica*).

**Methodology/Principal Findings:**

In a sample of 922 adult barn swallows from a single population breeding in Italy we found one very common (Q_7_) and three rare (Q_5_, Q_6_, Q_8_) length variants of a functionally significant polyglutamine repeat. Rare (2.9%) Q_7_/Q_8_ heterozygous females, but not males, bred significantly later than common (91.5%) Q_7_/Q_7_ females, consistent with the expectation that ‘long’ alleles cause late breeding, as observed in a resident population of another bird species. Because breeding date depends on arrival date from migration, present results suggest that the association between breeding date and *Clock* might be mediated by migration phenology. In addition, fecundity selection appears to be operating against Q_7_/Q_8_ because late migrating/breeding swallows have fewer clutches per season, and late breeding has additional negative selection effects via reduced offspring longevity. Genotype frequencies varied marginally non-significantly with age, as Q_7_/Q_8_ frequency showed a 4-fold reduction in old individuals. This result suggests negative viability selection against Q_7_/Q_8_, possibly mediated by costs of late breeding.

**Conclusions/Significance:**

This is the first study of migratory birds showing an association between breeding phenology and *Clock* genotype and suggesting that negative selection occurs on a phenologically deviant genotype. Low polymorphism at *Clock* may constrain microevolutionary phenological response to changing climate, and may thus contribute to the decline of barn swallow populations.

## Introduction

The ability to appropriately match the timing of critical life-history stages to temporal variation in ecological conditions is often under positive selection [1]–[2] (see also [3]). In environments where biotic factors and abiotic conditions oscillate periodically, organisms can greatly benefit from anticipating the advent of the best extrinsic conditions for key life history events such as emergence from immature developmental stages, migration and breeding. This is predicted to be especially the case when the pace of the shift in physiological and behavioural traits that precede such activities is slower than that of temporal changes in ecological conditions, or when spatio-temporal environmental heterogeneity produces abrupt changes in ecological conditions to which organisms are exposed. For example, migratory birds that breed in temperate or high latitude biomes with marked seasonal cycles in temperature and productivity need to schedule the onset of migration so as to arrive to the breeding grounds and exploit narrow peaks in food abundance weeks/months later, without the possibility to directly assess conditions at destination [4]–[7] (but see [8]).

Because seasonal variation in ecological conditions is determined by the astronomical position of the Earth relative to the Sun, which also causes circannual variation in the relative duration of day and night (photoperiod) at any given place on the Earth in a highly consistent way over millennia, photoperiod provides an accurate cue to optimally set the timing of annual life-history events. Indeed, photoperiod is the main cue that birds, for example, use in timing their migration and breeding [7], [9], [10].

Perception of variation in day length is in fact a major proximate mechanism beating the time of seasonal, periodic changes in physiology and behaviour of several organisms [10]–[13]. The ‘circadian clock’ senses temporal variation in light/dark cycles and produces a cascade of physiological processes that can ultimately cause adaptive behavioural shifts, such as flowering in plants, and preparing to and undertaking migration or breeding in birds [5], [10], [14], [15]. A large body of studies has led to the identification of several genes that are in control of the circadian clock and to the dissection of the molecular bases of circadian oscillations [15]. *Clock* gene networks have been characterized in organisms from fungi and plants to birds and mammals, and their functioning in producing biochemical circadian fluctuations shares similarities among phylogenetically distant taxa [16], [17].

All *Clock* genes encode elements of an auto-regulated transcriptional–translational feedback loop termed the ‘core circadian oscillator’ (CCO) [10], [14], [18]–[23]. Within CCO, *Clock* and *Bmal1* gene products heterodimerize to produce a transcription factor which constitutes both a positive driver of the molecular oscillations themselves and an ‘output’ signal from the circadian oscillator [24]–[26]. Studies of mice have demonstrated that an important role is played by the carboxyl-terminal polyglutamine repeat (poly Q) domain, observing that changes in the number of poly Q repeats affect circadian rhythms [27], [28].

Empirical evidence for a link between poly Q and phenology of migration and reproduction has partly originated from analyses of latitudinal variation in the poly Q repeat length [29]. In salmon species (*Oncorhynchus* spp.), timing of migration is under photoperiodic control and shows considerable geographical variation, as an adaptation to climate and river water flow regimes [28], [30]. Extensive population studies have demonstrated clinal variation in poly Q length as would be predicted by involvement of *Clock* in controlling the expression of migration according to ecological conditions. In the Chinook salmon *O. tshawytscha*, for example, *OtsClock1b* alleles increase in length with latitude across North American rivers [30]. In a partially migratory avian species, the blue tit (*Cyanistes caeruleus*), length of *Clk*polyQcds increases with latitude among 14 populations [31]. However, a study of migratory bluethroats (*Luscinia svecica*) yielded no evidence of clinal variation in allele length [31]. An analysis of resident populations of blue tits and great tits (*Parus major*) provided contrasting results over the role of *Clock* polymorphism in generating breeding phenological variation [32], [33]. Blue tit females, but not males, with fewer poly Q repeats bred earlier. However, in the closely related, syntopic great tit, there was considerably smaller genetic variation at *Clock* and absence of covariation with breeding phenology. A recent study of five distant barn swallow (*Hirundo rustica*) populations disclosed very low polymorphism at the *Clock* poly Q region [34]. Finally, a comparison among five *Tachycineta* swallow species found considerable variation in polymorphism at *Clock* poly Q but no evidence of latitudinal variation across species [35]. In addition, no evidence was found for an association between laying date and *Clock* genotype at the within-species level [35].

Despite long-standing interest of chronobiological studies in circadian oscillators, those mentioned above are the only, mostly very recent studies that have been published on the consequences of *Clock* gene polymorphism for the breeding phenology or migratoriness of birds [36]. On the other hand, there is evidence for genetically based variation and selection on both timing of migration and breeding in birds [7], [37]–[39], making the investigation of the genetic machinery controlling such phenological variation of utmost importance, particularly in a period when climate change is challenging the ability of populations to adapt to rapidly changing conditions [6], [7].

Because of the paucity of studies on birds and the contrasting evidence on the involvement of *Clock* in generating phenological variation, we analyzed breeding dates of barn swallows in relation to *Clock* genotype. Based on the evidence for blue tits [32] (but see [35]), we expected individuals with longer *Clock* alleles to breed relatively late. However, we expected this effect to be manifest only among females, as breeding dates are believed to be constrained by female physiological disposition to egg laying [32], [40].

In addition, we expected the frequency of *Clock* genotypes that dictate late breeding to be smaller among older individuals. This was predicted because selection mediated by the cost of reproduction may increase late in the season, as a result of worsening of conditions for breeding as the season progresses. We therefore compared the genotype frequencies between yearlings and older individuals.

## Materials and Methods

### Study Species

The barn swallow is a semi-colonial, socially monogamous passerine bird with biparental care of the progeny. Barn swallows breeding in Europe overwinter in sub-Saharan Africa and reach their breeding quarters in March-May. Yearlings arrive from migration and breed later than older individuals. Most females (91.2% in our study population) lay 4–6 eggs per clutch. Yearling females may lay one or two clutches per breeding season while older females normally lay two, seldom three clutches. Fledging success is very high, as 80–90% of the eggs produce a fledgling. Barn swallows are short-lived, with a life-expectancy at first breeding of ca. 1.3 years. In Europe, the large majority of barn swallows breeds synantropically in rural buildings or small villages, typically in farms with livestock [41]–[43].

### Field Procedures

During four breeding seasons (2002: n  =  173 individuals from 6 different farms; 2005: n  =  74, 7 farms; 2009: n  =  89, 4 farms, 2011: n  =  586, 19 farms) we studied barn swallows breeding at a total of 29 farms in an intensively cultivated farmland area near Milano (N Italy). We chose to consider birds from different, non-consecutive years in order to reduce the odds of stochastic extremeness both in breeding dates and gene frequencies, and to reduce any effect of temporal autocorrelation in the data. Owing to low levels of genetic diversity at *Clock*, in the analyses we pooled the data from different years. In the analyses, however, we also appropriately controlled for any effect of inter-annual variation in mean breeding dates due to e.g. meteorological conditions by including year as a random effect in linear mixed models (see *Statistical analyses*).

We regularly visited the colonies to record breeding events and capture breeding adults. Because adults normally spend the night inside the buildings where they breed, they were efficiently captured by placing mist nets at all entrances before dawn. All individuals were subjected to a number of standard measurements and a blood sample was collected by puncturing the brachial vein. Before being released, all individuals were marked with a unique combination of colour rings and patches on breast and belly feathers. Sex of adults was identified by inspection for a brood patch (indicating female, as males do not incubate in European populations) and for a prominent cloacal protuberance (indicating male), and by direct observation of sexual and social behaviour at the nest. In case of any doubt, sexing was confirmed molecularly [44]. For the purpose of the present study, we recorded Julian date of laying of the first egg in the first clutch for females or social mate of individual males. This variable was used as the core phenological indicator of timing of breeding throughout the study.

To obtain information on age of the breeding adults we took advantage of the extremely high breeding philopatry of barn swallows in our study area as well as in other parts of their European range, which implies that individuals that have bred in a particular farm in one year return to breed in the same farm in the following year(s) [41], [45], [46] (C. Scandolara pers. comm.). The farms for which information on age of breeding adults was available were those where we managed to capture the vast majority of the breeding adults in the year (*i*-1) preceding that (year *i*) to which individual phenological data refer. High breeding philopatry in combination with effectiveness of our effort to capture the vast majority of the breeding adults imply that adults that had not already been captured as adults during year *i*-1 in year *i* could be assumed to be yearlings hatched in year *i*-1. Conversely, individuals that had already been captured as adults in year *i*-1 were considered as two- or more years old. Thus, for the purpose of this study we classified swallows as yearlings (1-year old) or older (≥ 2 years old) birds [41], [45], [46]. The core sample of the present study consists of 632 adults for which both age and breeding date were known (see *Results* for age by sex sample sizes). Because breeding dates are strongly affected by age (see above) and the frequency of particular genotypes among older birds was very low (see below), the analyses were run on either age class separately. However, an additional 290 genotyped individuals of unknown age/breeding date were included in the statistics of allele frequencies and heterozygosity, and in the tests on deviation from Hardy-Weinberg equilibrium (HWE).

Most nestlings were ringed every year in the study farms, and we could thus confirm that no pairs of the very few siblings (see also below) that are recruited in their original colony were included in the sample. Indeed, barn swallows have very low natal philopatry, as only less than 5% of offspring were recruited locally on average. Hence, given: 1) the very low natal philopatry and large natal dispersal of barn swallows [41], [43]; 2) the very large size of the population of swallows breeding at a distance from the study colonies within the natal dispersal of barn swallows [47]; 3) that siblings seem not to disperse together (our unpublished data); and 4) that less than 40% of the swallows breeding in any given colony were included in the study, the chances that siblings that could not be recognized as such (because they had not been ringed as nestlings) were included in the analyses are likely to be very small. Moreover, due to large natal dispersal and point 4 above, the chances that parent-offspring pairs were included also appear to be very small and in no case this occurred for local recruits whose parents could be identified by direct observation at the nest.

### Analysis of *Clock* Gene

Total genomic DNA was extracted from blood samples collected from the brachial vein in heparinized capillary tubes. Blood was stored in a cool bag while in the field and subsequently at -20°C in the lab, until used. DNA extraction was performed by alkaline lysis using 6 µL of blood in 100 µL of a 50 mM NaOH at 100°C for 20 minutes. Extracted DNA was quantified using a spectrophotometer and diluted to a final concentration of 50–100 ng/µL [44].

In order to design species-specific primer for genotyping purposes, we initially used the primers published by [31] on barn swallow DNA samples. The amplified PCR fragments were then sequenced (BMR genomics, Padua, Italy) and a new set of primers designed on barn swallow genomic sequence. Genomic DNA samples were screened for length polymorphism in the *Clk*polyQcds by PCR amplification followed by resolution and detection on a conventional DNA sequencing machine using a commercial GeneScan service for genotyping (Macrogen Inc., Seoul, Korea). We used PCR forward primer (5′-labelled with 6-FAM dye) 5′-(6FAM)GGGACAGGTGGTGACAGCTTATC-3′ and reverse primer 5′- CTGCTGATGGTCCTGCTGACT-3′ (Sigma-Aldrich). Amplification reactions were as follows: 2 µL of extracted DNA (100–200 ng), 1.5 mM of Mg^2+^; Taq DNA polymerase 1.35 U (Genespin, Milano, Italy); 0.4 µL of each primer (stock 10 µM); 2 µL of dNTPs (stock 2mM), in a final volume of 20 µL. PCR amplification profile was as follows: 95°C/2 min, followed by 5 cycles at 95°C/30s, 65°C/30s 72°C/30s, decreasing 1°C each cycle (touchdown PCR), then by 30 cycles at 95°C/30 s, 60°C/30 s, 72°C/45 s. A final extension step was included at 72°C for 7 min. Samples were kept at 7°C until analyzed. Two internal size standards were used: the conventional molecular size standard HD400 ROX (Applied Biosystems) and a molecular weight standard generated ‘in-house’ by using a previously characterized swallow DNA sample, homozygous for the most common (Q_7_; see below) allele using the same forward primer, this time labeled with HEX. The PCR amplification with 6-FAM and HEX labeled primers were carried out separately and then 2 µL of the HEX-labeled PCR amplification reaction were added to each sample.

The reliability of molecular data were confirmed by randomly picking 80 samples and repeating the GeneScan analysis independently. Moreover each heterozygous genotype was double checked by repeating the PCR amplification reaction and sequencing. The most common allele was sequenced and its PCR amplification fragment proved to be 112bp long, coding for a CLOCK protein containing a stretch of 7 glutamine residues. In accordance with [31], the allele was named Q_7_ (or *Clk*polyQ_7_). Recently, the *Clock* gene sequence of the barn swallow has been published in GenBank [34]. Our sequences perfectly match those published (acc. no. JN642465-JN642524) The screening of 922 barn swallow individuals led to the identification of a total of 4 alleles, each differing by multiple of 3 base pairs. The alleles were named according to the number of glutamine residues predicted in the mature protein, as Q_5_-Q_8_ [34] Statistical tests were conducted using genotype classes.

### Statistical Analysis

We relied on linear mixed models to test for the effect of genotype on breeding date. Genotype was included in the analyses as a fixed factor. To control for any interannual variation in mean breeding dates and in their variances, we ran linear mixed models while including year as a random factor. The assumption of homogeneity of variances in breeding dates among genotypes within sex by age groups was always met (Levene test: P > 0.224). To check for stability of the results that were significant at these analyses of variance we applied non parametric (Kruskal-Wallis, Mann-Withney U) tests. Variation in genotype frequencies between age classes was tested by *G*-test while applying William’s conservative correction procedure [48]. These analyses were run with SAS 9.2 package (SAS Institute Inc, Boca Raton). Tests for deviation from HWE were performed using GenePop 4.0 package [49] while applying the following parameters in Markov chain analyses: dememorization: 10,000, batches: 10,000, interactions per batch: 10,000.

### Ethics Statement

Upon capture, barn swallows were kept in cloth bags in a safe position, as is standard practice in bird ringing studies. Blood samples were collected by puncturing the brachial vein. The puncturing site was accurately disinfected. All individuals were released as soon as possible, usually within 1 hour of capture. After being released, swallows behaved normally and observations at the nest on dozens of individuals confirmed that they resumed their normal breeding activities. The study was carried out under permission of the local authority (Regione Lombardia #M1.2002.0001646) responsible for authorizing animal studies in the wild. The farmers gave permission to enter their properties (list of farmers available from the authors upon request). No approval from an ethical committee was required for this study.

## Results

We genotyped 478 females and 444 males. The most common of the four alleles in the population (Q_7_) accounted for 96.7% of the overall allelic diversity (Table 1). Allele frequencies and observed heterozygosity were very similar in either sex (Table 1).

**Table 1:** Allele frequencies and observed heterozygosity (H) for the *Clock* gene in 922 adult barn swallows.

	*N*	Q_5_	Q_6_	Q_7_	Q_8_	H
Total	1844	0.05	2.49	96.58	0.87	0.066
Males	888	0.11	2.82	96.17	0.90	0.072
Females	956	0	2.20	96.97	0.84	0.061

*n*  =  number of alleles.

The observed genotype frequencies did not deviate from HWE (P  =  0.185; Table 2). Splitting genotype frequencies by age revealed a significant deviation from HWE among older (P  =  0.015), but not among yearling individuals (P > 0.99). Among older individuals, more Q_6_/Q_8_ and fewer Q_7_/Q_8_ than expected were recorded (Table 2). However, separate analyses of the four sex by age groups showed that only older males significantly deviated from HWE (P  =  0.008; other groups: P always > 0.99).

**Table 2:** Observed and expected (after Levene’s correction) genotype frequencies of the *Clock* gene for 922 adult barn swallows.

Genotype	Observed	Expected	Yearlings	Older
Q_5_/Q_7_ *	1	0.97		
Q_6_/Q_7_	44	44.45	19 (6, 13)	18 (9, 9)
Q_6_/Q_8_	2	0.40	0 (0, 0)	2 (0, 2)
Q_7_/Q_7_	861	860.06	312 (165, 147)	282 (153, 129)
Q_7_/Q_8_	14	15.46	10 (6, 4)	2 (1, 1)

* The age of the single Q_5_/Q_7_ individual was not known. The genotypes not reported had observed frequency of 0 and expected frequency ≤ 0.57 in all cases. Total genotype frequencies of the 645 of known age (yearlings or older) are also reported. In parentheses, the frequency of females or males is detailed.

**Figure 1:**
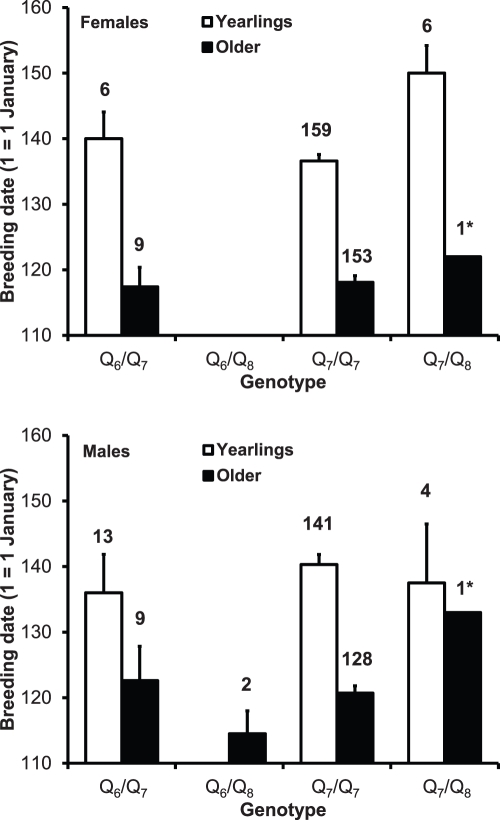
Mean (+ s.e.) breeding date of the genotypes in the sex by age classes of the barn swallow. Numbers in the body of the graphs are sample sizes. Breeding date data of the single Q_7_/Q_8_ older female or male (denoted by 1*) are reported for completeness but are not included in the statistical analyses. The difference between yearling female Q_7_/Q_7_ and Q_7_/Q_8_ was significant (P < 0.05, see Results).

Breeding date was known for 632 genotyped individuals (171 yearling and 163 older females; 158 yearling and 140 older males). Some genotypes were not represented in some of the four age by sex classes or their absolute frequency was 1. The analyses of breeding date therefore had to be run on each class separately, because an unbalanced design with missing data in some age by sex classes prevented testing the interaction effects among genotype, age and sex. In a mixed model with year as a random effect, mean breeding date significantly varied according to genotype among yearling females (F_2,166.4_  =  3.43, P  =  0.035; Fig. 1). Q_7_/Q_8_ heterozygotes bred on average 13 days later than Q_7_/Q_7_ homozygotes and 10 days later than Q_6_/Q_7_ heterozygotes, with a significant difference between Q_7_/Q_8_ and the homozygotes (LSD test; P  =  0.019), whereas the other pairwise differences were non-significant (P ≥ 0.247). It should be noted, however, that the test comparing Q_7_/Q_8_ and Q_6_/Q_7_ had low power owing to the small frequency of both heterozygotes. No significant difference in breeding date existed between Q_6_/Q_7_ and Q_7_/Q_7_ among older females (F_1,157.7_  =  0.15, P  =  0.902; the single Q_7_/Q_8_ female was excluded; Fig. 1). In addition, in mixed models breeding date did not vary among male genotype classes (yearlings: F_2,153.3_  =  0.43, P  =  0.655; older: F_2,136_  =  0.33, P  =  0.721, the single Q_7_/Q_8_ older male was excluded; Fig. 1). Kruskal-Wallis non-parametric analysis of variance confirmed a significant variation in breeding dates among the three yearling female groups (χ^2^  =  6.52, df  =  2, P  =  0.038), while Mann-Whitney U test confirmed the difference between Q_7_/Q_8_ and Q_7_/Q_7_ (Z  =  2.47, P  =  0.014).

The frequency of the four genotypes showed a marginally non-significant variation with age (G-test with William’s correction, G_3_  =  7.27, P  =  0.064). In particular, the relative frequency of Q_7_/Q_8_ individuals was reduced among older compared to yearling individuals (2.9% vs. 0.7%; Table 2). The patterns of reduction in Q_7_/Q_8_ frequency were similar in either sex, though not statistically significant (Table 2; males: G_3_  =  4.33, P  =  0.228; females: G_2_  =  4.22, P  =  0.121). However, when we analyzed variation in frequencies by contrasting Q_7_/Q_8_ against the other genotypes pooled, a significant difference between the two age classes emerged (G_1_  =  4.83, P  =  0.028). Finally, Q_6_/Q_8_ that did not occur among yearlings was conversely found among older individuals, although at very low frequency ( =  0.007), possibly due to random sampling error of this very rare genotype.

## Discussion

Circadian clocks are major mechanisms that allow organisms to sense photoperiod and ultimately produce temporal shifts in physiology and behaviour [14], [15], [17]. While there is detailed information on the phylogenetic distribution of *Clock* genes and the molecular and genetic functioning of circadian oscillators, there are relatively few studies of the phenotypic consequences of variation at genes associated with circadian clocks in the wild, both in terms of phenology and viability [32], [33], [36].

The present study contributes to filling this gap of knowledge, and shows that in a barn swallow population *Clock* gene diversity is correlated to detectable variation in breeding phenology of females. This statistically significant evidence emerged despite small frequency of the phenologically deviant, late breeding genotype and the fact that considerable environmental variation is generated in bird breeding dates by meteorological and climatic conditions [50]–[52]. A major implication of this finding is that negative fecundity selection operates via reduced number of clutches per breeding season. This effect is additive to the negative consequences of hatching date on offspring longevity (see below).

Diversity at the *Clock* poly Q region has been hypothesized to result from balancing selection caused by spatio-temporal ecological heterogeneity [31]. Clinal variation in poly Q allele length may be stabilized by adaptation to local patterns of circannual variation in photoperiod, whereby different alleles allow to adaptively ‘interpret’ and accommodate rates of change in photoperiod, which depend on latitude [31], [32]. Length of poly Q alleles has been shown to increase with latitude in the blue tit and also in the Chinook salmon [30], [31], and this cline is possibly maintained by selection for locally optimal timing of breeding or spawning. Reciprocally, different *Clock* genotypes may produce different phenology when exposed to the same local photoperiod, owing to a different interpretation of the same light-dark cycle. The core finding of our study, that Q_7_/Q_8_ females heterozygous for the longer alleles delayed breeding relative to Q_7_/Q_7_ homozygotes, is thus consistent with the hypothesis of a direct involvement of *Clock* in breeding phenological variation. Three pieces of evidence specifically corroborate this interpretation. First, the association between allele length and breeding date is in the same direction as reported for blue tits [32]. Second, the sign of the association is consistent with the observed latitudinal increase in *Clock* allele length in other species [31], whose migration/breeding dates also increase with latitude [42]. Finally, the association was observed among females but not males, consistent with the expectation that any genetic control of breeding date should be more easily uncovered in females [53].

The previous studies of bird breeding phenology in relation to *Clock* diversity concerned two resident populations of blue or great tits [32], [33] and five species of swallows of the genus *Tachycineta* [35] with diverse migratory habits, and provided contrasting results over the association between breeding date and *Clock*. A covariation was detected in resident blue tits [32] but not e.g. in an analysis of migratory *Tachycineta bicolor* swallows where the effects of age on breeding date could be controlled for [35]. All European barn swallow breeding populations are migratory [41], [42]. Breeding date in our study population as well as in other European populations may be constrained by arrival date, as there is a very strong, positive association between these variables within age-classes, and some individuals arrive after early breeding ones have already started laying. In a sample of yearlings for which arrival date could be estimated accurately, the correlation coefficient was as high as 0.60 (n  =  128, P < 0.0001; regression coefficient  =  0.460 (0.054 s.e.)) [41]. Hence, our results are compatible with the interpretation that *Clock* controls the photoperiodic responses that govern the course of spring migration from Africa, thereby affecting breeding phenology. A potential role of *Clock* in controlling migration has been advocated for Pacific salmon and also suggested to produce latitudinal variation in migratoriness of tit populations [30], [31], [54]. We have no accurate information on arrival dates of most of the swallows in our breeding population because obtaining estimates of arrival date is extremely time-consuming. However, the regression coefficient of breeding on arrival date, which was smaller than 1 (see above), indicates that the spread in arrival dates across genotypes may be even larger than that of breeding dates [55]. Admittedly, this argument is indirect and not conclusive, as it is possible that Q_7_/Q_8_ females arrived at the same time as Q_7_/Q_7_ ones, and then postponed breeding.

The difference in breeding phenology between Q_7_/Q_7_ and Q_7_/Q_8_ yearling females was found despite the small number of Q_7_/Q_8_ heterozygotes that emerged from an otherwise large sample. On the other hand, the test comparing mean breeding dates of Q_6_/Q_7_ and Q_7_/Q_7_ was also based on a small sample of Q_6_/Q_7_ females. We therefore refrain from drawing conclusions from the non-significant result of the latter test. Even more vulnerable to type II statistical error was the comparison between breeding dates of Q_6_/Q_7_ and Q_7_/Q_8_ yearling females, that was based on 6 data points per genotype. In addition, extreme rarity of Q_7_/Q_8_ older females ( =  1 out of 163 for which breeding date was known) prevented any analysis of the effect of this genotype on breeding date among older females, which breed earlier than yearlings. It should also be noted that genotype-by-environment interactions could have resulted in differential patterns of variation in the effect of *Clock* genotype on breeding date between years (and thus cohorts) due to, for example, inter-annual variation in ecological conditions at the breeding grounds. Such potential effects could not be tested here explicitly because of low levels of polymorphism at *Clock*.

Indeed, a striking feature of the population we studied is its small genetic diversity at *Clock*. The most frequent allele (Q_7_) accounted for 96.6% of the total allelic diversity, only 4 different alleles were observed, and heterozygosity was consequently very low. These findings are consistent with those recently reported in a study of barn swallows from five distant geographical populations [34]. In that study, Q_7_ was found to account for 93.9% or more of the alleles in all populations. Contrary to the present study, no Q_5_ was detected, although this could be due to either differences in sampling effort, to geographic variation in allele frequencies, or both. The other reports of *Clock* genetic diversity published to date for wild bird populations have disclosed a bewildering difference even among closely related species or cospecific populations [31]–[33], [35], [36]. For example, even syntopic blue and great tits hugely differed in allele diversity and heterozygosity, with blue tits showing larger diversity [31], [33]. The genetic profile of the population we studied resembles that of a great tit population, which was dominated by a single allele with a frequency of 95.7% [33]. Moreover, *Clock* polymorphism was found to considerably vary among five species of swallows of the genus *Tachycineta*, although there was no hint that genetic diversity was related to latitude [35].

Important functional roles of *Clock* have been advocated for diverse organisms and are also suggested by its high level of phylogenetic conservation [14], [17]. Because breeding date has been consistently shown to be under selection in birds [39], [41], [56], reduced allelic diversity and the evidence of breeding delay associated with one of the rare alleles argue for a role of selection. Indeed, we gathered both direct and indirect evidence suggestive of selection against Q_7_/Q_8_. First, genotype frequencies varied between age classes, with a marked decrease in the frequency of Q_7_/Q_8_ among two- or more years old individuals compared to yearlings, suggesting that the *Clock* genotype that is associated with late breeding in females, also caused reduced viability. Second, by delaying breeding by ca. 13 days ( =  *a*), Q_7_/Q_8_ females suffer reduced seasonal breeding success. In our study population, yearling females may lay one or two clutches per season. However, the frequency of second clutches declines with date of laying of the first clutch, by ca. 0.012 ( =  *b*) per day. Combining the information on frequency of second clutches among yearling females (*c*  =  0.45–0.56), mean fecundity in first (*d*  =  4.5 eggs) or second (*e*  =  3.7) clutches, and assuming negligible change in clutch size over a 13 days period, constancy of inter-clutch interval, and neglecting rare third clutches, Q_7_/Q_8_ females are expected to suffer a 4–5% ( =  {(*d* + (*c* * *e*) – (*a* * *b* * c *e))/(*d* + (*c* * *e*))} * 100) reduction in annual fecundity. However, variation of breeding date associated with age was larger than that among yearling females’ *Clock* genotypes (see Fig. 1), implying that variance in breeding success due to age effects largely exceeds that due to *Clock* genotype effects. Moreover, in our study population there is a strong negative effect of hatching date on longevity after sexual maturity (i.e. after 1 year of age) [57], such that an increase in breeding date by 13 days causes reduction in longevity by ca. 0.2 years. Hence, delayed breeding results in a fitness cost mediated by offspring longevity in addition to that via seasonal fecundity.

An open question remains as to which mechanisms generate a reduction in the frequency of Q_7_/Q_8_ with age. As far as females are concerned, it can be speculated that delayed breeding may entail a survival cost, if reproductive investment is larger for individuals that breed late, when ecological conditions are less favourable [58]. Moreover, females that breed earlier have longer inter-clutch interval and enjoy higher survival [59]. If Q_7_/Q_8_ also dictates different migration schedules, an additional possibility is that survival costs arise during migration. At present, the causes of any larger mortality of Q_7_/Q_8_ males are obscure. However, the patterns of reduction in frequency of Q_7_/Q_8_ with age were similar in either sex.

An additional question is what maintains allele polymorphism and particularly Q_8_ alleles in the population, even in the face of apparently negative viability and fecundity selection against Q_7_/Q_8_. The possibility that temporal variation in selection may balance gene frequencies cannot be ruled out. In particular years, severely adverse weather conditions at the time when first clutches are normally laid by yearling females may set a selective advantage for late breeding birds. Alternatively, gene flow may maintain Q_8_, though at low frequency. While breeding dispersal of barn swallows is very small, mean natal dispersal is in the order of kilometers [43]. However, variance is large, with some yearlings being recruited hundreds of kilometers away from their natal site [43]. In addition, natal dispersal may be under-estimated because of spatial heterogeneity in sampling effort. If latitudinal variation in optimal timing of breeding maintains *Clock* polymorphism across European populations, low frequencies of Q_8_ may be caused by immigration of yearlings from populations breeding at higher latitudes, although lack of among-populations variation in *Clock* polymorphism at coarse inter-continental scale may argue against this hypothesis [34]. Finally, Q_8_ alleles could be maintained at low frequency in the population through mutation of the most common allele.

Selection on *Clock* and low levels of polymorphisms have a bearing for the interpretation of the mechanisms that mediate dramatic negative population trends in this species and also to predict its evolutionary fate. The barn swallow is among the farmland birds that have suffered the largest recent decline, which in our study area has been of 8.4% per year over 10 years [60], [61]. The decline of barn swallows may partly be attributed to changes in agricultural/farming practices in their breeding quarters or in ecological changes in staging and wintering areas, although conclusive evidence is still missing [61]–[63]. However, the decline of bird breeding populations can also be attributed to their inability to keep track of the consequences of climate change on timing of spring phenological events by advancing their breeding dates [6], [64], [65]. Low genetic polymorphism at *Clock* may thus entail small potential for adaptive evolutionary response by locally adapted breeding populations under climate change. On the other hand, it is also entirely possible that low polymorphism at *Clock* genes itself is the product of adaptation of the barn swallow population we studied to current climate change.

In conclusion, this is the second study providing evidence for an association between *Clock* genotype and timing of breeding in any wild bird population, and the first concerning a long-distance migrant. Because breeding date is strongly positively correlated with date of arrival from migration, the link between *Clock* and breeding phenology may be mediated by an effect of *Clock* on migration. The increase in breeding date with the number of poly Q repeats is consistent with previous observations of a latitudinal increase in frequency of long alleles in two other vertebrate species, because breeding date increases with latitude. Low levels of polymorphism in *Clock*, combined with age-related variation in frequency of *Clock* genotypes and with the negative effect of late breeding on annual fecundity and progeny longevity suggest ongoing selection on *Clock* in the population we studied.
