# Correction: *Clock* Gene Variation Is Associated with Breeding Phenology and Maybe under Directional Selection in the Migratory Barn Swallow

**DOI:** 10.1371/annotation/b738de1b-6b12-4f1b-9736-7d7e0be5c0da

**Published:** 2012-05-31

**Authors:** Manuela Caprioli, Roberto Ambrosini, Giuseppe Boncoraglio, Emanuele Gatti, Andrea Romano, Maria Romano, Diego Rubolini, Luca Gianfranceschi, Nicola Saino

There is a formatting error in the title. The correct title is: *Clock* Gene Variation Is Associated with Breeding Phenology and May Be under Directional Selection in the Migratory Barn Swallow.

